# EMR usability and patient safety: a national survey of physicians

**DOI:** 10.1038/s41746-025-01657-4

**Published:** 2025-05-15

**Authors:** David Schwappach, Wolf Hautz, Gert Krummrey, Yvonne Pfeiffer, Raj M. Ratwani

**Affiliations:** 1 Institute of Social and Preventive Medicine (ISPM), University of Bern, Bern, Switzerland; 2 Department of Emergency Medicine, Inselspital University Hospital, University of Bern, Bern, Switzerland; 3 Institute for Medical Informatics (I4MI), Bern University of Applied Sciences (BFH), Biel/Bienne, Switzerland; 4 harmfree healthcare, Wädenswil, Switzerland; 5 Institute of Nursing Science (INS), Faculty of Medicine, University of Basel, Basel, Switzerland; 6 MedStar Health National Center for Human Factors in Healthcare, Washington, DC, USA; 7 Georgetown University School of Medicine, Washington, D.C, USA

**Keywords:** Outcomes research, Translational research

## Abstract

Despite widespread adoption of electronic medical records (EMRs), concerns persist regarding their usability and implications for patient safety. This national cross-sectional survey assessed physicians’ perceptions of EMR usability across safety-relevant domains. Among 1933 respondents from diverse care settings, 56% reported that their EMR did not enhance patient safety, and 50% perceived their system as inefficient. Usability ratings averaged 52% of the maximum score. Statistically significant differences were observed between EMRs in outpatient (η² = 0.13) and hospital (η² = 0.37) settings. Multilevel modeling attributed 38% of the variance in usability ratings to differences between EMRs, 51% to hospital-level variation within EMRs, and 11% to physician-level differences. Canonical discriminant analysis identified key differentiating usability features, including system response times, excessive alerts, prevention of data entry errors, and support for collaboration. These findings underscore substantial limitations in current EMR systems and reinforce the value of comparative usability assessments to inform targeted improvements in digital health infrastructure.

## Introduction

In the United States, the 2009 Health Information Technology for Economic and Clinical Health (HITECH) Act promoted the widespread adoption of electronic medical records (EMRs) with an approximate $40B federal investment. The widespread adoption of EMRs marked a new era of ‘digitized healthcare,’ often associated with promises and expectations of greater efficiency and enhanced patient safety. However, multiple studies conducted over the past decade have shown that EMRs are associated with clinical errors, delayed care delivery, and serious patient harm^1–4^. EMR usability, which is focused on the ease of interaction between clinician and computer interface, measured through efficiency, effectiveness, and satisfaction^5^ has emerged as a critical factor for patient safety and clinician burnout. In a survey study among US physicians, better EMR usability was associated with lower task load and lower odds of burnout^6^. A strong dose-response relationship between EMR usability and the odds of burnout was observed in both nurses and physicians^7,8^. Several federal and private sector efforts have been put in place to promote improved EMR usability in the US, including modifications to the federal certification program, greater transparency on the usability of EMR products, and support to EMR developers to improve their usability and safety practices. More recently, large US EMR developers have expanded to European countries. Studies suggest that usability and safety challenges persist, although national data remain scant. In scenario-based usability assessments, a large variation in error rates in performing routine clinical tasks has been observed^9^. In that study, error rates ranged from 27% to 40% between two EMRs widely used in Switzerland, measured across two hospital implementations. For some scenarios, the error rate exceeded 50%. To assess EMR usability, the system usability scale (SUS) has been widely used^10^. The SUS is a ten-item Likert scale that covers different aspects of system usability. Bloom et al. applied the SUS to emergency medicine physicians across the UK^11^. The median SUS score across EMRs and organizations was 53 of the maximum score of 100, with a range of 35–65. In a similar study, Fuhrmann et al. surveyed ophthalmologists in Germany^12^. The mean EMR SUS score was 66, with a range of 32–87 between different systems.

Systematic usability assessments on the national level can be used to compare EMR systems, to monitor developments over time, and to detect subgroups of clinicians who may have specific needs (either personal or due to the tasks they perform), and to target improvements. In Switzerland, as in many European countries, many hospitals currently transition from first-generation EMRs to more advanced systems, which presents a timely opportunity to establish a baseline assessment of EMR usability and inform the choice of new systems. The primary aim of this cross-sectional study was thus to evaluate the perceived usability of EMRs across key domains, including workflow integration, navigation, and cognitive load, in a nationwide sample of physicians. We applied a novel survey instrument to assess usability with a focus on patient safety. Specifically, we sought to assess variation across EMRs and work settings and to identify whether specific patterns of usability ratings are associated with different EMRs.

## Results

In total, 1933 physicians using an EMR completed the survey. The survey response rate was 9.5%, and the survey completion rate was 87.7% of those who started the survey. Table 1 reports participants’ characteristics. Half of the respondents (50.0%) reported having over two years of experience with their EMR, followed by 18.4% with 1–2 years, 17.4% with 6–12 months, 8.1% with 3–6 months, and 6.1% with less than three months. Most respondents (48.0%) had received less than 4 h of training for their EMR, and 29.6% received 4–8 h. Smaller proportions received 9–16 h (8.4%) or more than 16 h (5.7%), or no training at all (8.3%). More than half of respondents (54.7%) assessed their proficiency with the EMR as advanced, while 29.4% considered themselves intermediate. A smaller proportion (13.3%) identified as experts, and 2.6% reported having problems working with the system.

**Table 1:** Respondents’ characteristics

	*n* (%)
*N*	1933
Age	
<35 years	690 (39.3%)
35–44 years	493 (28.1%)
45–54 years	322 (18.4%)
55–64 years	201 (11.5%)
>64 years	48 (2.7%)
Gender	
Female	973 (55.9%)
Male	769 (44.1%)
Medical specialty	
General internal medicine	682 (35.3%)
Anesthesiology	116 (6.0%)
Surgery	87 (4.5%)
Gynecology and obstetrics	82 (4.2%)
Intensive care medicine	55 (2.8%)
Cardiology	46 (2.4%)
Pediatrics and adolescent medicine	121 (6.3%)
Medical oncology	22 (1.1%)
Nephrology	24 (1.2%)
Neurology	46 (2.4%)
Orthopedic surgery	40 (2.1%)
Psychiatry and psychotherapy	104 (5.4%)
Rheumatology	23 (1.2%)
Urology	23 (1.2%)
Other*	462 (23.9%)
Work setting	
Medical practice	495 (25.6%)
Hospital	1438 (74.4%)
Medical Role	
Medical student	46 (2.4%)
Resident	690 (35.9%)
Senior physician	435 (22.6%)
Consultant	98 (5.1%)
Hospital specialist	45 (2.3%)
Lead physician	212 (11.0%)
Chief physician	56 (2.9%)
Visiting consultant	97 (5.0%)
Other*	243 (12.6%)
Type of hospital (for hospital physicians)	
University hospital	375 (28.2%)
Cantonal hospital	458 (34.5%)
Regional hospital	344 (25.9%)
Psychiatric clinic	56 (4.2%)
Rehabilitation clinic	28 (2.1%)
Other specialty clinic	68 (5.1%)
Hospital department (for hospital physicians)	
Anesthesiology	114 (8.6%)
Surgery, incl. subspecialties	161 (12.2%)
Gynecology and obstetrics	54 (4.1%)
Internal medicine, incl. subspecialties	514 (38.8%)
Intensive care medicine	56 (4.2%)
Emergency department	115 (8.7%)
Pediatrics	82 (6.2%)
Psychiatry and psychotherapy	82 (6.2%)
Other*	147 (11.1%)
Type of practice (for outpatient physicians)	
Solo practice	105 (23.3%)
Dual/group Practice	345 (76.7%)
Specialization of practice (for outpatient physicians)	
Primary care	217 (47.8%)
Specialized care	157 (34.6%)
Primary and specialized care	80 (17.6%)

*other summarized over detailed categories.

Physicians evaluated 24 different EMRs in practice settings and 16 EMRs in hospital settings. The mean number of survey responses per EMR vendor was 19.0 in the practice setting and 88.3 in the hospital setting. Six practice EMRs and ten hospital EMRs received more than 29 ratings each.

**Fig. 1:**
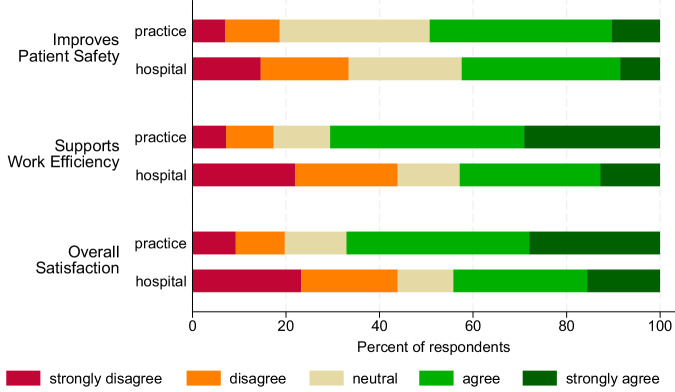
Distribution of responses to global EMR satisfaction items by work setting. This figure displays the distribution of physicians’ responses to three items assessing perceived safety, efficiency, and overall satisfaction with electronic medical records (EMRs), stratified by work setting (hospital vs. medical practice). Responses are presented as percentages across Likert-scale categories for each item and setting. The three items constitute the **S**afety-**E**fficiency-**S**atisfaction (SES). Full item wording is provided in Supplementary Table 1.

Most physicians felt the EMR did not improve safety (55.9%), and 50.1% felt it did not support efficient work. Overall, 50% of respondents reported satisfaction with their EMR (Fig. 1). The mean SES (**s**atisfaction, **e**fficiency, **s**afety) scale score across EMRs was 2.9 (SD 1.3) among hospital and 3.6 (SD 1.1) among practice physicians (*p* < 0.001) with higher scores indicating more positive evaluations. There were considerable differences in mean SES scores between evaluated EMRs in both settings (hospital EMRs with >29 ratings: *p* < 0.001; Eta squared = 0.37, CI 0.34–0.41; practice EMRs with >29 ratings: *p* < 0.001; Eta squared = 0.10, CI 0.03–0.15).

**Fig. 2:**
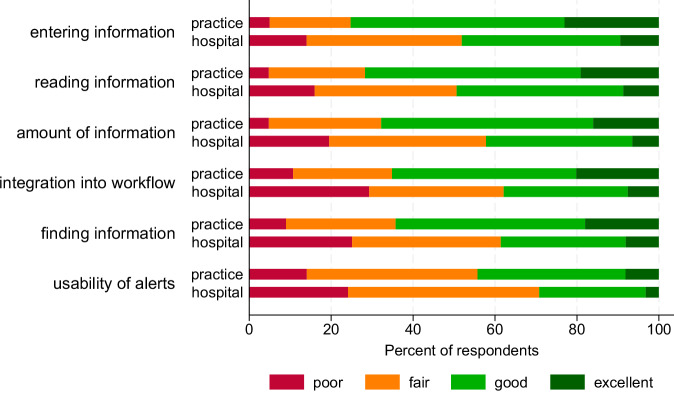
Distribution of responses to the Hendrix et al.^29^ EMR usability scale by work setting. This figure presents the distribution of physicians’ responses to the 6 items of the usability scale developed by Hendrix et al., stratified by work setting (hospital vs. medical practice). Responses are shown as percentages across Likert-scale categories for each item. Differences in response patterns highlight variations in user experience across settings. Item wording is available in Supplementary Table 1.

**Fig. 3:**
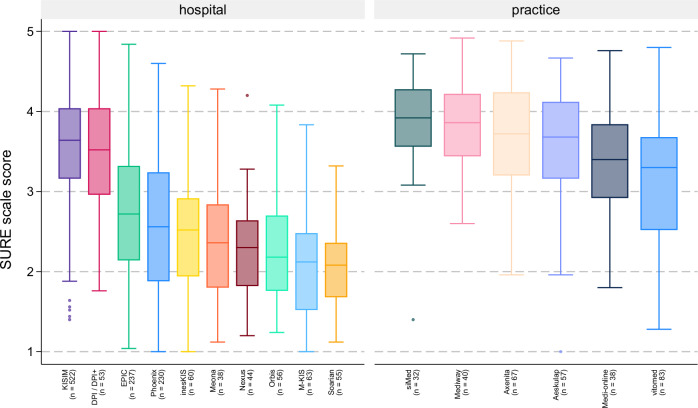
SURE scale scores for EMRs by work setting. This figure shows boxplots of SURE (**S**ystem **U**sability and **R**isk **E**valuation) scale scores for electronic medical record (EMR) systems with more than 29 physician ratings, stratified by work setting (hospital vs. medical practice). Scores range from 1 to 5. Boxplots indicate median, interquartile range, and outliers. Only EMR systems meeting the threshold for subgroup comparison are included. ANOVA revealed significant differences across EMRs in both settings.

Figure 2 presents the responses to the six items of Hendrix’ usability scale (HEX) by work setting. The mean scale score over all items of the Hendrix usability scale was 2.3 (SD 0.75) for hospital physicians and 2.7 (SD 0.69) for physicians in the medical practice (*p* < 0.001). The same three items (integration into workflow, finding information, and usability of alerts) received the highest number of ‘poor’ ratings among hospital and practice physicians. The SURE (**s**ystem **u**sability and **r**isk **e**valuation) and HEX scales were highly correlated (*r* = 0.91, CI 0.90–0.92), demonstrating very good criterion validity of SURE. McDonald’s omega scale reliability coefficient for the new 25-item SURE scale was 0.96, indicating excellent internal consistency. Table 2 lists all SURE items by care setting. On average, respondents rated their EMR at 52.2% of the maximum possible score (POMP). Across care settings, remembering how to use the system, time wasted by the EMR, and collaboration with internal colleagues received the highest mean levels of agreement (SURE items 10, 24, and 15). The lowest mean levels of agreement were observed in supporting collaboration with external colleagues, prioritizing daily tasks, and highlighting potential data entry errors (SURE items 16, 17, and 23). There were substantial differences in the ratings of physicians from hospital and practice settings, with EMRs in the practice setting achieving considerably higher mean SURE scores (3.52 vs. 2.95, *p* < 0.001). Among EMRs with >29 ratings, there were significant differences in mean SURE scores between EMRs, both within the practice (*p* < 0.001, Eta squared = 0.13, CI 0.06–0.19) and the hospital setting (*p* < 0.001, Eta squared = 0.37, CI 0.32–0.40) (Fig. 3). The three-level hierarchical model including 556 observations, 7 different EMRs and 18 hospital sites revealed that 37.9% of the total variance in SURE usability scores was attributable to differences between EMRs (ICC = 0.38, 95% CI: 0.13–0.72), 51.3% was attributable to differences between hospitals within EMRs (ICC = 0.51, 95% CI: 0.28–0.74), and the remaining 11% (ICC = 0.110, 95% CI: 0.035–0.250) was due to individual-level variation among physicians.

**Table 2:** SURE (**s**ystem **u**sability and **r**isk **e**valuation) items and summary measures by care setting

	Work setting								
	Medical practice			hospital			Total		
	Mean	SD	% agree	Mean	SD	% agree	Mean	SD	% agree
SURE_1: MySystem helps me work efficiently.	3.75	1.17	69.12	2.88	1.38	41.88	3.10	1.38	48.69
SURE_2: MySystem supports me in providing good care for the patients.	3.72	1.09	65.55	3.22	1.22	48.14	3.34	1.21	52.50
SURE_3: MySystem makes it easy to make good decisions in treatment/care.	3.21	1.11	39.79	2.92	1.13	32.82	2.99	1.13	34.56
SURE_4: MySystem helps to prevent errors in care.	3.20	1.15	44.00	2.89	1.22	39.08	2.96	1.21	40.30
SURE_5: MySystem provides a useful overview of a patient’s current health condition.	3.34	1.22	53.26	3.01	1.31	45.55	3.09	1.30	47.48
SURE_6: MySystem integrates well into our processes, such as generating reports.	3.71	1.19	68.07	3.16	1.35	50.21	3.30	1.33	54.68
SURE_7: MySystem is intuitive to use.	3.56	1.20	60.93	2.77	1.40	36.76	2.96	1.40	42.82
SURE_8: I find the display and labels on the screen in MySystem easy to understand.	3.91	1.05	75.58	3.29	1.27	53.86	3.45	1.25	59.30
SURE_9: I find it easy to navigate in MySystem.	3.86	1.15	71.13	3.07	1.37	44.44	3.27	1.36	51.12
SURE_10: I can easily remember how to use MySystem.	4.10	1.01	80.25	3.53	1.25	59.60	3.67	1.22	64.77
SURE_11: The arrangement and sequence of fields and functions on the screen in MySystem.	3.78	1.06	70.91	3.02	1.28	43.51	3.21	1.28	50.37
SURE_12: Information on the screen in MySystem is clearly visible (e.g., windows or drop-down menus).	3.74	1.14	69.85	3.12	1.29	48.23	3.27	1.29	53.64
SURE_13: MySystem fits well with my way of working.	3.62	1.21	63.66	2.89	1.36	38.73	3.07	1.36	44.95
SURE_14: In MySystem, I can quickly find the information about a patient that I need at the moment.	3.74	1.16	69.03	2.98	1.28	42.06	3.17	1.29	48.79
SURE_15: MySystem supports collaboration with internal colleagues.	3.73	1.11	64.13	3.38	1.27	56.88	3.47	1.24	58.67
SURE_16: MySystem supports collaboration with external colleagues.	3.14	1.14	37.80	2.35	1.18	18.68	2.55	1.22	23.44
SURE_17: MySystem helps me prioritize my daily tasks.	3.21	1.17	42.58	2.46	1.16	20.34	2.65	1.21	25.90
SURE_18: I understand how MySystem generates its recommendations, such as scores or alerts.	2.88	1.16	29.22	2.85	1.19	33.74	2.86	1.18	32.62
*SURE_19: Useless alerts in MySystem often interrupt my workflow.	2.33	1.22	18.44	3.06	1.24	39.70	2.88	1.28	34.37
*SURE_20: Long loading times in MySystem significantly hinder my work.	2.57	1.40	29.72	3.33	1.43	52.14	3.14	1.46	46.52
SURE_21: MySystem supports my decision-making rather than dictating it.	3.12	1.02	30.65	2.96	0.99	26.64	3.00	1.00	27.64
SURE_22: Incorrect data can be easily corrected in MySystem.	3.76	1.14	68.76	3.11	1.20	46.08	3.27	1.22	51.77
SURE_23: MySystem highlights potential errors in data entry (e.g., missing decimal point or unrealistic body weight).	2.64	1.24	26.74	2.85	1.20	34.21	2.80	1.21	32.33
*SURE_24: Inefficiency of MySystem makes me waste a lot of time each day unnecessarily.	2.84	1.35	36.23	3.73	1.27	64.01	3.50	1.35	57.04
*SURE_25: Working in MySystem is very exhausting.	2.31	1.17	16.92	3.05	1.30	41.00	2.87	1.31	34.96
SURE scale score	3.52	0.81		2.95	0.92		3.09	0.93	
POMP (Percent of Maximal Possible)	62.90	20.20		48.68	22.97		52.23	23.14	

*Negatively coded items were reverse-coded for mean and POMP scale scores.

**Fig. 4:**
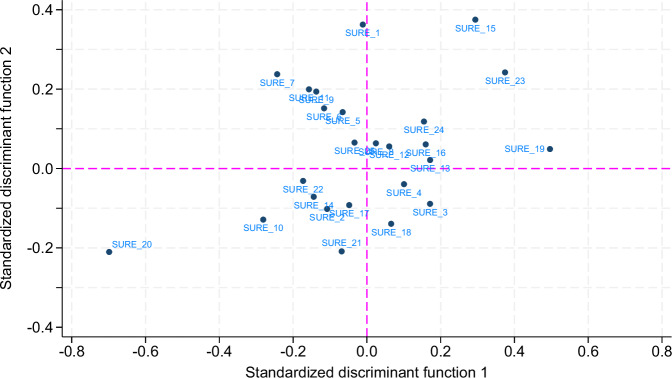
Canonical discriminant function plot based on SURE scale items. This figure illustrates the results of a canonical discriminant analysis using physician responses to the System Usability and Risk Evaluation (SURE) items. Function 1 accounts for 76% of the variance and primarily separates EMR EPIC from the other systems. Function 2 explains 24% of the variance and distinguishes EMR Phoenix. The analysis highlights key items that differentiate EMR systems based on physician-reported data.

The discriminant function analysis on the three most common hospital EMRs (*n* = 989) identified two highly significant discriminant functions with canonical correlations of 0.76 and 0.54, respectively (Wilks’ lambda was significant overall and for both functions, with *p* < 0.001). Together, these functions accounted for all variance in group separation, with function 1 explaining 76% and function 2 explaining 24%. The group centroids on the canonical discriminant functions demonstrated clear separation among the three EMR groups. For function 1, the centroids were 0.84 for EMR Phoenix, −2.03 for EMR EPIC, and 0.57 for EMR KISIM. For function 2, the centroids were −1.08 for EMR Phoenix, −0.11 for EMR EPIC, and 0.53 for EMR KISIM. These results suggest that EMR EPIC is distinct from the others based on function 1, while EMR Phoenix is most distinct on function 2. To understand which aspects of EMRs drive these distinctions, we examined the items most strongly associated with each function: Key contributors to function 1 included loading times (SURE item 20), useless alerts (item 19), prevention of data entry errors (item 23) and ease of collaboration with internal colleagues (item 15). For function 2, the most relevant items were collaboration with internal colleagues (item 15), support for working efficiently (item 1), loading times (item 20), and prevention of data entry errors (item 23) (Fig. 4). The classification analysis indicated that the discriminant functions correctly classified EMRs in 80% of cases, demonstrating strong predictive accuracy.

Post-hoc analyses revealed significant differences in mean SES and SURE scores among physician subgroups (Table 3). Anesthesiologists and intensive care physicians provided substantially lower ratings regarding EMR usability and satisfaction. Longer experience with EMR use was strongly associated with higher usability and satisfaction ratings, particularly after surpassing a threshold of one year of use. Additionally, EMR training was significantly related to SES and SURE scores; physicians who reported no or minimal training, or those with more than 16 h of training, provided notably lower ratings on both scales.

**Table 3:** Differences in SURE (**s**ystem **u**sability and **r**isk **e**valuation) and SES (**S**afety, **E**fficiency, **S**atisfaction) mean scores by participant characteristics

	Mean (SD)	
	SURE	SES
Age	(*p* = 0.011)	(*p* = 0.143)
<35 years	3.02 (0.94)	3.06 (1.29)
35–44 years	3.08 (0.89)	3.14 (1.22)
45–54 years	3.10 (0.98)	3.12 (1.25)
55–64 years	3.19 (0.91)	3.26 (1.20)
>64 years	3.44 (0.84)	3.42 (1.07)
Gender	(*p* = 0.553)	(*p* = 0.984)
Female	3.07 (0.89)	3.13 (1.23)
Male	3.10 (0.97)	3.12 (1.26)
Medical specialty	(*p* < 0.001)	(*p* < 0.001)
General internal medicine	3.18 (0.90)	3.22 (1.24)
Anesthesiology	2.66 (0.88)	2.64 (1.20)
Surgery	3.24 (1.00)	3.39 (1.27)
Gynecology and obstetrics	3.24 (0.85)	3.34 (1.17)
Intensive care medicine	2.73 (0.83)	2.72 (1.23)
Cardiology	2.94 (1.08)	2.89 (1.40)
Pediatrics and adolescent medicine	3.00 (0.87)	3.05 (1.19)
Medical oncology	3.01 (0.84)	3.08 (1.17)
Nephrology	3.11 (0.94)	3.04 (1.31)
Neurology	2.90 (0.98)	2.92 (1.31)
Orthopedic surgery	3.00 (1.06)	3.00 (1.40)
Psychiatry and psychotherapy	3.01 (0.91)	3.02 (1.16)
Rheumatology	3.56 (0.91)	3.72 (1.06)
Urology	2.85 (1.05)	2.83 (1.43)
Other*	3.13 (0.92)	3.08 (1.25)
Work setting	(*p* < 0.001)	(*p* < 0.001)
Medical practice	3.52 (0.81)	3.58 (1.07)
Hospital	2.95 (0.92)	2.95 (1.27)
Type of hospital (for hospital physicians)	(*p* < 0.001)	(*p* < 0.001)
University hospital	2.76 (0.88)	2.74 (1.24)
Cantonal hospital	3.20 (0.90)	3.34 (1.21)
Regional hospital	2.91 (0.91)	2.91 (1.26)
Psychiatric clinic	2.72 (0.80)	2.64 (1.11)
Rehabilitation clinic	2.40 (0.71)	2.12 (0.95)
Other specialty clinic	2.51 (0.97)	2.39 (1.31)
Type of practice (for outpatient physicians)	(*p* = 0.147)	(*p* = 0.135)
Solo practice	3.61 (0.82)	3.72 (1.07)
Dual/group Practice	3.48 (0.80)	3.54 (1.07)
EHR duration of use	(*p* < 0.001)	(*p* < 0.001)
<3 months	2.94 (0.86)	2.94 (1.17)
3–6 months	2.93 (0.96)	2.94 (1.22)
6–12 months	2.84 (0.87)	2.79 (1.24)
1–2 years	3.06 (0.94)	3.10 (1.27)
>2 years	3.20 (0.92)	3.28 (1.23)
EHR training	(*p* = 0.015)	(*p* = 0.023)
None	3.01 (0.96)	3.04 (1.26)
<4 h	3.06 (0.92)	3.07 (1.24)
4–8 h	3.16 (0.92)	3.25 (1.24)
9–16 h	3.11 (0.91)	3.17 (1.25)
>16 h	2.85 (0.97)	2.88 (1.34)

*other summarized over detailed categories.

*p*-values indicate the results of one-way analysis of variance.

## Discussion

Today, the choice between different available EMRs is often only possible based on claims by their developers. Comparative research directly informing the choice is rare. This study compared clinician assessments of EMR usability in a Swiss national sample. Overall, a large sample of physicians did not agree that their EMR supports safe and efficient patient care, with hospital physicians providing significantly and substantially poorer ratings. It is alarming that nearly two-thirds of hospital physicians indicated that EMR inefficiency wastes their time each day, and only forty percent agree that their EMR helps to prevent errors in care – a failure in the two key promises of digitalization of healthcare. Across both settings and all products, EMRs achieved only slightly over 50% of the maximum possible usability score. While these figures are highly disappointing, they are well in accordance with studies using more generic instruments ^11,12^. Ratings provided by outpatient physicians in our study are notably similar to the responses obtained in a recent survey among US family physicians, both in terms of satisfaction and usability using the same instrument^13^. More generally, these results suggest there has been no substantial improvement in EMR usability and safety over the last 15 years. Our results indicate that the newly developed SURE instrument is internally consistent and strongly associated with existing measures. In our sample, a relatively small number of items were highly effective in discriminating three widespread EMRs in Switzerland. This information can be used to generate strength and weakness profiles of the various EMRs or to identify and prioritize areas for improvement.

While none of the evaluated EMRs performed well, some performed substantially worse than others. The large effect sizes seen in our outcome measures between EMRs clearly suggest that physicians do not indiscriminately provide poor ratings but rather respond to the actual performance of each EMR. Most notably, the specific implementation of an EMR in each hospital seems to play a vital role in how users perceive its usability. This finding aligns with prior research showing that, for example, the number of mouse clicks required to complete identical tasks varied across hospital implementations of the same EMR^9^. The large variance in usability scores explained by hospital sites emphasizes the role of the hospital IT department in adapting EMRs to their clinician user’s needs and to support them in daily practice. Thus, accountability cannot simply and solely be shifted to developers. Rather, resources to establish respectful collaborations between clinician users and hospital IT, as well as between vendors and hospital IT, are required to develop, test, and implement local improvements of EMR systems and rigorously evaluate their effects on usability and patient safety. Research confirms that such targeted usability interventions can yield positive outcomes: For example, Mazur et al. investigated the effects of a usability enhancement to the EMR test result organization and reported decreased cognitive workload and increased performance in management of abnormal test results^14^. Bakker et al. showed that reducing and tailoring potential drug-drug-interaction alerts to a specific setting significantly reduced the number of administered high-risk drug combinations^15^.

Our results also provide some insights into groups of physicians reporting particularly low usability and satisfaction with the EMR: Those new to a system and those working in complex, fast-paced clinical environments, namely, intensive care physicians and anesthesiologists, including those working in the ED. Both relationships also help to explain why hospital physicians, compared to office-based physicians, provided significantly lower usability ratings. These findings confirm results from largely different healthcare systems, like the US and Scandinavia. For example, a longitudinal repeated assessment of physicians’ perceptions showed a marked drop in the perceived safety of the system during an EMR transition phase, which did not return to pre-transition values^16^. Recently, Lohmann-Lafrenz et al. observed very poor perceived usability six months after go-live of a comprehensive, sector-wide, patient-centered EMR in Norway^17^. In this study, poor usability was also associated with higher rates of burnout, insomnia, and turnover intentions. These findings point to a broader systemic trend: contemporary EMR systems being implemented are of such complexity and demand that, even after extended periods of use, many physicians report limited perceived support for their clinical work.

A major strength of our study is the use of a national sample of physicians with a large variety of work settings, medical roles, and specialties. Physicians were asked by their professional organization and not their employer, which might have increased trust and willingness to report safety-related EMR performance. We used robust methodology to develop a new usability assessment instrument that allows comparison of EMRs across specific aspects and provides insights into strengths and weaknesses and areas for improvement. Our study has some limitations: The response rate was relatively low, although this is not uncommon given the chosen study design and participant recruitment approach. Since information regarding personal characteristics of physicians using EMRs was unavailable, we cannot confirm the representativeness of our sample. Compared to the general population of physicians practicing in Switzerland, participants in our sample were more likely to be female, younger, and employed in hospital settings (https://aerztestatistik.fmh.ch/). Additionally, irrespective of these demographic characteristics, a response bias towards the “unsatisfied” cannot be ruled out^18^. A limitation of our multilevel analysis is the inclusion of EMRs with only very few hospital sites. This may confound EMR- and hospital-level effects, potentially inflating the variance attributed to EMR products. This limitation should be considered when interpreting the variance decomposition. Finally, comparing results between home-grown and commercial products could provide valuable insights; however, such information was not consistently available for all EMRs included in the study. Moreover, this distinction itself may lack precision, as some EMRs initially developed as home-grown solutions and only later transitioned into commercial products, while others, although commercially developed, have undergone extensive local adaptations, further complicating clear categorization.

EMR usability and patient safety continue to be rated poorly in a national physician survey, highlighting the need for substantial and targeted improvements of EMRs. Comparing different EMRs on detailed usability criteria can help to foster transparency, benefiting developers, healthcare organizations, and clinician users. In publicly funded healthcare systems, very large investments in new EMR systems should be accompanied by strict evaluations, and clinician-perceived usability should be one crucial parameter.

## Methods

This study was designed as a national cross-sectional survey study.

### Survey instrument

The survey instrument was developed in an iterative procedure, in which the level of detail and length were continuously balanced. In a first step, items were pooled and clustered from existing instruments based on a review of the literature^16,19–28^. Additional items were developed to address specific patient safety-related usability aspects that were insufficiently covered in existing instruments, such as support for internal collaboration, based on our prior research. The preliminary item set was tested in a read-and-think-aloud experiment with 15 diverse clinicians using videoconferencing and recording for later in-depth review. The adapted survey was then field tested in three smaller pilot tests with office-based and hospital physicians (total *n* = 200). With the data obtained, basic psychometric tests such as analysis of door- and ceiling effects, missing values, variation, internal consistency, and correlation were performed. Comments were analyzed for direct feedback on items. Some items were revised for comprehension and wording, others were discarded.

The final survey consisted of: a list of EMRs used in Switzerland from which respondents were asked to select their currently used system; level of EMR expertise and training received; a set of 25 items assessing clinician perceptions of patient safety-related EMR usability across several domains, e.g., integration into workflows, navigation, cognitive load, alerts (SURE: **s**ystem **u**sability and **r**isk **e**valuation); three global rating items assessing overall **s**atisfaction with the used EMR, overall system **e**fficiency, and overall impact on patient **s**afety (SES); the six item usability scale published by Hendrix et al.^29^ was integrated for validation purposes (HEX); personal and workplace characteristics, e.g., hospital type. The 28 SURE and SES usability items used a 5-point Likert scale asking for the level of agreement with statements. HEX items use a four-point scale (poor to excellent). Within the survey, the name of the EMR was dynamically replaced with the system used by each respondent. To avoid ordering effects, items were randomly rotated within scales. Supplementary Table 1 lists all survey items.

### Participants

The sample consists of members of the Swiss Association of Resident and Attending Physicians, excluding retired members and dentists. Membership in the association is voluntary. The association represents physicians from across all regions of the country working in a wide range of healthcare settings. A total of *n* = 20,245 persons were invited (roughly 50% of all practicing physicians in Switzerland).

### Data collection

The survey was administered online in German and French in December 2024 using SoSci Survey^30^. Participants were invited by email with individual access codes. Two reminders were sent to non-participants after one and three weeks. For data protection reasons, all emails and access codes were sent by the association’s membership office.

### Ethics

The study was exempt from review by the lead ethics commission of the Canton of Bern (Req-2024-01052). Participation was voluntary, and informed consent was obtained from all respondents.

### Statistical analysis

We used descriptive statistics to summarize response data using means and standard deviations (SD) for continuous variables, stratified by setting (hospital vs. medical practice). For the SURE scale, we computed both mean scale scores and the percent of maximum possible score (POMP), a transformation that is easier to interpret. POMP = [(*observed* − *minimum*)/(*maximum* - *minimum*)] × 100, where observed = the observed score for a single case, *minimum* = the minimum possible score on the scale, and *maximum* = the maximum possible score on the scale^31^. POMP always ranges between 0 and 100. For example, with the 25-item SURE and a sum-score range of 25–125, a sum-score of 25 equals 0%, 75 equals 50%, and 125 equals 100%. Negatively coded items were reverse-coded before mean and POMP scale scores were computed. Internal consistency of the scales was assessed using McDonald’s omega, a more general measure of reliability, as Cronbach’s Alpha, but with a similar interpretation^32^. As an indicator of criterion validity, the SURE mean scores were correlated with Hendrix’ usability scale (HEX) ^29^. ANOVA was conducted to test the ability of the SURE and SES scales to discriminate between different EMRs and across institutions. Only EMRs with at least 30 ratings were included in this analysis. Eta squared was calculated as a measure of effect size. To estimate the proportion of variance in SURE scores attributable to differences between EMRs, we conducted a multilevel analysis using a three-level hierarchical model, with physicians nested within hospitals, and hospitals nested within EMRs, and no other additional variables. Using the name of EMR, hospital type, and postcode, we identified clusters of participants from the same hospital site. The intraclass correlation coefficients (ICCs) were calculated to quantify the proportion of variance in SURE scores attributable to each hierarchical level. Canonical linear discriminant function analysis was performed to identify those SURE survey items that contribute most to EMR differentiation. Canonical linear discriminant function analysis (LDA) is a multivariate statistical technique used to differentiate predefined groups - in this case, EMRs - by identifying linear combinations of predictor variables (SURE items) that maximize the separation among these groups. Each canonical discriminant function represents a unique dimension of variation, with “Function 1” accounting for the greatest possible discrimination between groups, followed by subsequent functions (e.g., “Function 2”), which explain progressively less variance and are statistically uncorrelated with prior functions. This analysis produces group centroids, defined as the mean discriminant function scores for each EMR category. Centroids thus represent each EMR group’s average or central location within the discriminant function space, facilitating visual and statistical interpretation of how distinctly the EMRs differ across these derived dimensions. This analysis was restricted to the three most common hospital EMRs with *n* > 200 to ensure model stability. A 2-sided *P* < 0.05 was considered statistically significant. All analyses were performed with STATA v18^33^.

## Supplementary information

Supplementary information

## Data Availability

The dataset analyzed in the study is not publicly available due to the sensitive nature of the data and the potential for deductive disclosure. Participants were assured of strict confidentiality, particularly with respect to their employers. Given the combination of variables such as hospital type, EMR system, medical role, and specialty, there exists a risk that individual physicians could be indirectly identified. Sharing such data would violate the ethical assurances provided to participants and our institutional review board's requirements.
