# Supplementary material for: EMR usability and patient safety: a national survey of physicians
Source: NPJ Digit Med. 2025 May 15;8:282. doi: 10.1038/s41746-025-01657-4 (PMC12081653; doi:10.1038/s41746-025-01657-4)
Supplement: Supplementary file 1 — Supplementary information [file 41746_2025_1657_MOESM1_ESM.pdf]

Supplementary Table 1: Survey items (English translated from German original)

Response scale: 5-point Likert from strongly disagree to strongly agree, unless otherwise indicated. MySystem is a placeholder which was dynamically replaced by the system used by the user (as selected from the presented list of systems).

**Name**	**Item text**
SURE	Now it's about your experiences with MySystem:<br>Do you agree with the following statements?
SURE1	MySystem helps me work efficiently.
SURE2	MySystem supports me in providing good care for the patients.
SURE3	MySystem makes it easy to make good decisions in treatment/care.
SURE4	MySystem helps to prevent errors in care.
SURE5	MySystem provides a useful overview of a patient's current health condition.
SURE6	MySystem integrates well into our processes, such as generating reports.
SURE7	MySystem is intuitive to use.
SURE8	I find the display and labels on the screen in MySystem easy to understand.
SURE9	I find it easy to navigate in MySystem.
SURE10	I can easily remember how to use MySystem.
SURE11	The arrangement and sequence of fields and functions on the screen in MySystem are logical.
SURE12	Information on the screen in MySystem is clearly visible (e.g., windows or drop-down menus).
SURE13	MySystem fits well with my way of working.
SURE14	In MySystem, I can quickly find the information about a patient that I need at the moment.
SURE15	MySystem supports collaboration with internal colleagues.
SURE16	MySystem supports collaboration with external colleagues.
SURE17	MySystem helps me prioritize my daily tasks.
SURE18	I understand how MySystem generates its recommendations, such as scores or alerts.
SURE19*	Useless alerts in MySystem often interrupt my workflow.
SURE20*	Long loading times in MySystem significantly hinder my work.
SURE21	MySystem supports my decision-making rather than dictating it.
SURE22	Incorrect data can be easily corrected in MySystem.
SURE23	MySystem highlights potential errors in data entry (e.g., missing decimal point or unrealistic body weight).
SURE24*	Inefficiency of MySystem makes me waste a lot of time each day unnecessarily.
SURE25*	Working in MySystem is very exhausting.
HEX	How would you assess the following usability dimensions of MySystem?<br>*(Response scale: poor / fair / good / excellent)*
HEX1	Entering information
HEX2	Reading information
HEX3	Amount of information
HEX4	Integration into workflow
HEX5	Finding information
HEX6	Usability of alerts
SES	Do you agree with the following statements?
SES1	Overall, MySystem improves patient safety.
SES2	Overall, I can work efficiently with MySystem.
SES3	Overall, I am satisfied with MySystem.
	How would you assess your own level of proficiency when working with MySystem?<br>*(Response options: expert / advanced / intermediate / I have difficulties)*
	How long have you been working with MySystem?

	(Response scale: < 3 months / 3 – 6 months / 6 – 12 months / 1 – 2 years / > 2 years)
	How much training have you received for MySystem in total?<br>*(Response scale: None / < 4 hours / 4 – 8 hours / 9 – 16 hours / > 16 hours)*
